# The role of the integument with respect to different modes of locomotion in the Nematalycidae (Endeostigmata)

**DOI:** 10.1007/s10493-014-9857-0

**Published:** 2014-10-30

**Authors:** Samuel J. Bolton, Gary R. Bauchan, Ronald Ochoa, Christopher Pooley, Hans Klompen

**Affiliations:** 1 Acarology Laboratory, Department of Evolution, Ecology and Organismal Biology, The Ohio State University, 1315 Kinnear Rd., Columbus, OH 43212 USA; 2 Electron and Confocal Microscopy Unit, USDA, ARS, BARC-West, Bldg. 012, 10300 Baltimore Ave., Beltsville, MD 20705-2350 USA; 3 Systematic Entomology Laboratory, USDA, ARS, BARC-West, Bldg. 005, 10300 Baltimore Ave., Beltsville, MD 20705-2350 USA

**Keywords:** Peristalsis, Striations, Anal valves, LT-SEM, Videography

## Abstract

Previous research on the locomotion of the Nematalycidae has only been undertaken on *Gordialycus*, which is by far the most elongated genus of the family. *Gordialycus* is dependent on an unusual form of peristalsis to move around. It was not known whether the genera of Nematalycidae with shorter bodies also used this mode of locomotion. Our videographic recordings of *Osperalycus* did not reveal peristalsis. Instead, this mite appears to move around the mineral regolith via the expansion and constriction of the metapodosomal and genital region, allowing greater versatility in the way that the annular regions contract and extend. This type of locomotion would enable relatively short bodied nematalycids to anchor themselves into secure positions before extending their anterior regions through tight spaces. Low-temperature scanning electron micrographs show that the short bodied genera have integumental features that appear to be associated with this mode of locomotion. Peristalsis is almost certainly a more derived form of locomotion that is an adaptation to the unusually long body form of *Gordialycus*.

**Electronic supplementary material:**

The online version of this article (doi:10.1007/s10493-014-9857-0) contains supplementary material, which is available to authorized users.

## Introduction

**Fig. 1:**
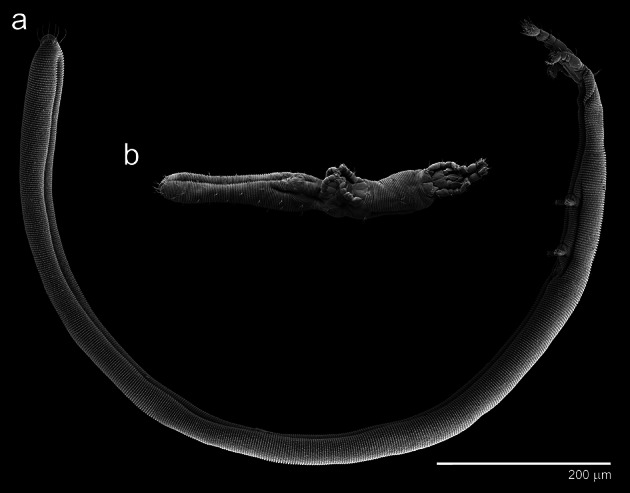
Two species of Nematalycidae. **a** *Gordialycus* sp. A (nymph/adult). **b** *Cunliffea strenzkei* (adult female)

The Nematalycidae is an unusual and cosmopolitan family of vermiform mites that inhabit mineral regolith (sands and mineral soil horizons). So far only five genera (all monospecific) have been described (Bolton et al. 2014; Coineau et al. 1967; Cunliffe 1956; Schubart 1973; Strenzke 1954). Despite the small number of species in this family, they contain a large amount of morphological variation with respect to several features, including the mouthparts (Bolton et al. submitted) and relative body length (Fig. 1).

The Nematalycidae possess several idiosyncratic features that pertain to their locomotion: the length of the body is lined with annular striations (annuli); legs I–II are separated from legs III–IV (the metapodosoma) by a long anterior region of the body; the genitalia are positioned directly behind the metapodosoma; finally, Nematalycidae possess a long posterior region behind the genitalia.

These highly elongated mites move around like worms by extending and contracting their bodies. The soft and flexible region of the integument between successive annuli folds or bends when the associated body region contracts, forcing the annuli together. Conversely, when the integument between successive annuli straightens, the annuli push apart and the associated body region extends. The contraction of a body region is induced by muscles that underlie the integument. The extension of the body occurs where longitudinal muscles relax. Although extension may be facilitated by some degree of elasticity in the integument (Haupt and Coineau 1999), any large degree of extension requires the generation of hydraulic pressure in the haemocoel via the constriction of another region of the body. Like other mites, the Nematalycidae lack circular muscles for reducing the body diameter (Haupt and Coineau 1999).

The annuli are lined with flat projections known as palettes, which are orientated so that their edges are perpendicular with respect to the annuli. These palettes have also been found in the Nanorchestidae (Alberti et al. 1981). Their function is not certain, but it has been hypothesized that they may be used to form a plastron to help avoid drowning (Haupt and Coineau 1999; Rounsevell and Greenslade 1988). It is likely that the Nematalycidae also (or instead) use them to improve the traction of the integument during movement (Coineau et al. 1978; Haupt and Coineau 1999).

Knowledge of the locomotion of the Nematalycidae has been based solely on studies of *Gordialycus*, an exceptionally elongated genus that can move via a form of peristalsis (Coineau and Coineau 1979; Coineau et al. 1978; Haupt and Coineau 1999). Given the lack of circular muscles for reducing the body diameter, peristalsis in *Gordialycus* only involves longitudinal muscles (Haupt and Coineau 1999; see the section of the discussion on anal valves for a more a detailed explanation).

The peristaltic motion of *Gordialycus* may only be suited to its unusually long body shape. We therefore undertook a comparative study of integumental characters associated with locomotion in the Nematalycidae using both light microscopy and low-temperature scanning electron microscopy (LT-SEM). The study was based on specimens of *Cunliffea strenzkei* Cunliffe, cf. *Psammolycus* sp. n., *Osperalycus tenerphagus* Bolton and Klompen, and *Gordialycus* spp. n. A–B. In addition, the locomotion of live specimens of *O. tenerphagus* was filmed. The videos of the live specimens were used to help interpret how integumental features observed under LT-SEM are used in locomotion. LT-SEM images of the other genera were examined to determine whether they shared some of these integumental features and, therefore, probably a similar mode of locomotion.

## Materials and methods

### LT-SEM

LT-SEM was carried out at the US Department of Agriculture, Electron and Confocal Microscopy Unit (Beltsville, MD, USA), using a S-4700 field emission scanning electron microscope (Hitachi High Technologies America, Pleasanton, CA, USA). Images were captured and processed using the techniques described in Bolton et al. (2014). Specimens were collected via soil washing in accordance with Kethley (1991). The technique was modified for collecting live specimens of *O. tenerphagus* for LT-SEM (see videography section below). All other species were collected in accordance with the original protocol and then stored in 80–95 % alcohol until they were mounted for LT-SEM.

Five species were collected and observed using LT-SEM: *Gordialycus* spp. n. A, B; *O. tenerphagus*; cf. *Psammolycus* sp. n.; and *C. strenzkei*. *Gordialycus* sp. A and B are the same as *Gordialycus* sp. A and B in Bolton et al. (in preparation). *Osperalycus tenerphagus* was collected from a silty clay loam in Columbus, Ohio. The other species were collected from sands in Indiana, Florida and California. Vouchers of all taxa are deposited at the Ohio State University Acarology Collection. Specimens were prepared for LT-SEM using the techniques described in Bolton et al. (2014).

### Videography

Live specimens of *O. tenerphagus* were collected using a modification of Kethley’s soil flotation method (Kethley 1991). The specimens were removed from floated material that had not yet been sieved. The floated material was placed into a Petri dish and observed under a dissection microscope. Any live specimens, if present, were then delicately lifted out. Some were placed on a small chunk of silty clay loam from their native soil and filmed using a stereomicroscope (Nikon SMZ-1500) equipped with a digital camcorder (Sony HDR-HC7). Note that the interstitial pores of this type of soil are too small to allow the passage of mites; *O. tenerphagus* must instead be living within the cracks and voids.

Other live specimens were placed directly in oil immersion fluid (Zeiss immersol 518N) and very gently mounted on a conventional slide underneath a circular coverslip (10 mm diameter). The mites were then filmed using a Zeiss Axioskop™ equipped with a phase contrast optical system and a digital camcorder (same make and model as above).

## Results and description

### Integument and idiosomal elongation (LT-SEM)

The degree of elongation of the idiosoma varies dramatically throughout the Nematalycidae because the length varies much more than the width (Fig. 1; Table 1). *Gordialycus* (idiosomal length >1.0 mm) is much longer than the two shortest genera—cf. *Psammolycus* and *Cunliffea*.

**Table 1:** Principal morphological features pertaining to locomotion

Genus	Adult idiosomal length (mm)	Adult idiosomal width (mm)	Protrusion of the anal valves	Shape of palettes	Longitudinal striations on the metapodosoma
*Gordialycus*	>1.0	0.05	Strong	Circular	Absent
*Osperalycus*	0.6	0.04	Strong	Circular	Present
*Cunliffea*	0.4	0.05	Weak	Pointed (blunt)	Present
cf. *Psammolycus*	0.3	0.03	Weak	Pointed (sharp)	Present

**Fig. 2:**
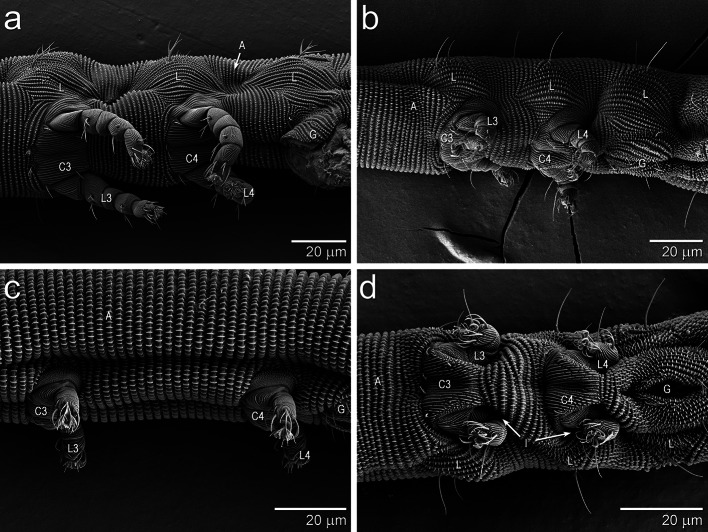
Lateral, lateroventral and ventral views of the metapodosomal and genital region—the anterior end is left in all images. **a** *Cunliffea strenzkei*. **b** *Osperalycus tenerphagus.* **c** *Gordialycus* sp. A. **d** *O. tenerphagus*—showing legs contracted into the metapodosoma. L, patch of longitudinal striations; A, annular striations; G, genital region; L3, leg 3; L4, leg 4; C3, coxal field 3; C4, coxal field 4; I, Indentation due to the contraction of the legs into the body

All of these taxa have an integument that is similar to the one described for *Gordialycus* (Haupt and Coineau 1999). Much of the integument of Nematalycidae consists of annuli. However, each side of the body of *Osperalycus*, *Cunliffea* and cf. *Psammolycus* has three small patches of integument with longitudinal striations (Fig. 2a, b: L). All three patches are located in the metapodosomal and genital region: two of the patches are directly above legs III and IV; the other is above the genitalia. Legs III and IV have enlarged coxal fields (Fig. 2a, b: C3, C4). Note that although the coxal fields have longitudinal striations, their striations are not lined with palettes, and they appear to be comprised of the same type of relatively rigid integument that comprises the standard leg segments. It is therefore unlikely that the integument of the coxal fields is able to fold.

The patches of longitudinal striations are absent in *Gordialycus* (Fig. 2c). Instead, the annuli curve around legs III and IV. Legs III and IV, including their coxal fields, are also greatly reduced in *Gordialycus* compared to the other genera. In one specimen of *Osperalycus*, legs III and IV were shown to be able to contract very tightly into the body, causing an indentation of the integument (Fig. 2d: I).

**Fig. 3:**
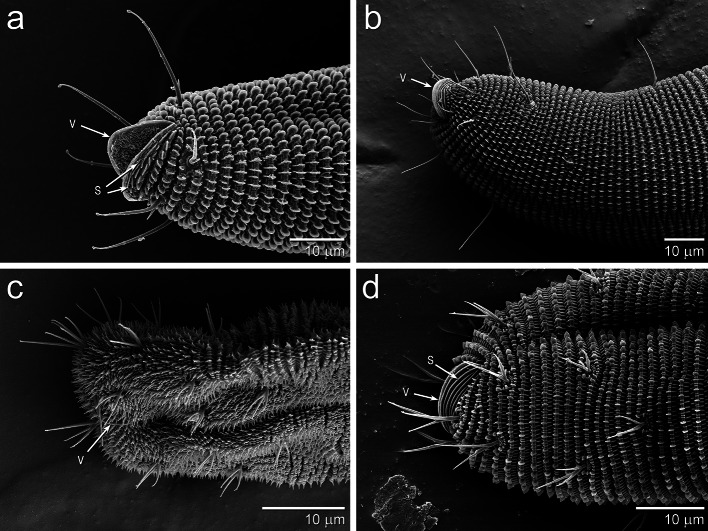
Ventrolateral views of the opisthosomal tip, showing the anal valves. **a** *Gordialycus* sp. B. **b** *Osperalycus tenerphagus.* **c** cf. *Psammolycus* sp. n.—anal valves barely discernible. **d** *Cunliffea strenzkei*. V, anal valve; S, striation without palettes

**Fig. 4:**
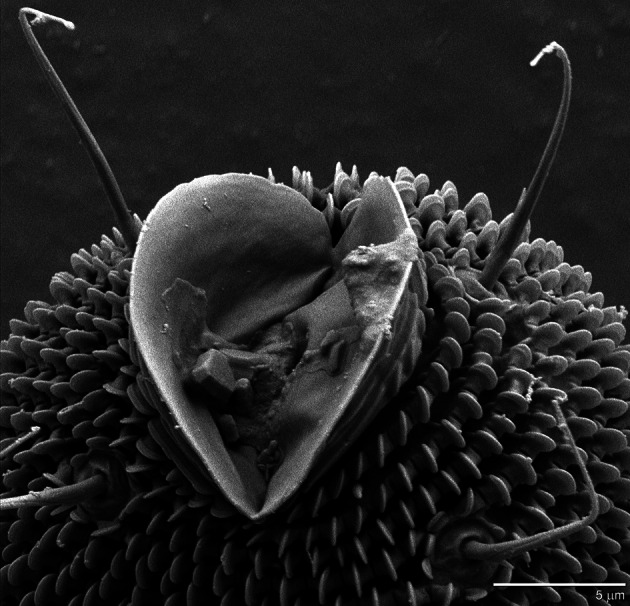
Close-up view of the anal valves of *Gordialycus* sp. A

**Fig. 5:**
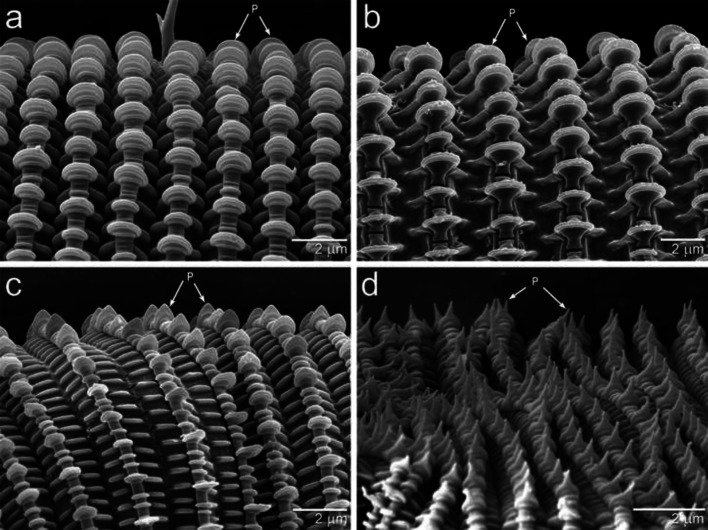
Palettes and integument. **a** *Osperalycus* *tenerphagus.* **b** *Gordialycus* sp. B. **c** *Cunliffea strenzkei*. **d** cf. *Psammolycus* sp. n. P, palette

In all five species, anal valves project from the anus (Figs. 3, 4). The external surface of the anal valves is lined with unpaletted striations that are similar to those found on other parts of the mite, including the legs and prodorsum. The palettes are rounded in *Gordialycus* and *Osperalycus* (Fig. 5a, b: P), whereas in *Cunliffea* and cf. *Psammolycus* they taper to a point. In *Cunliffea*, each palette tapers to a relatively short, blunt point (Fig. 5c). By contrast, each palette of cf. *Psammolycus* consists of a long, narrow projection, tapering to a sharp point (Fig. 5d: P).

### Locomotion (videography)

Late nymphal and adult instars of *Osperalycus* would often move around by pushing off against the tip of their opisthosoma (the location of the anal valves). The posterior region of the opisthosoma initially bends and contracts along its longitudinal axis. The tip of the opisthosoma is then pushed into the soil, anchoring it in place. The posterior region of the opisthosoma subsequently extends and straightens, pushing the center and front of the mite forwards (Online Resource 1). This occurs via several distinct waves of extension that proceed anteriorly from the anus. As this is happening, the mite is also crawling forwards using its legs. Once the posterior region is fully extended, the tip of the opisthosoma detaches from the soil substrate. This movement mechanism was also occasionally observed in the protonymph (Online Resource 2). However, the posterior region of the opisthosoma is more frequently dragged around by the protonymph (Online Resource 3).

**Fig. 6:**
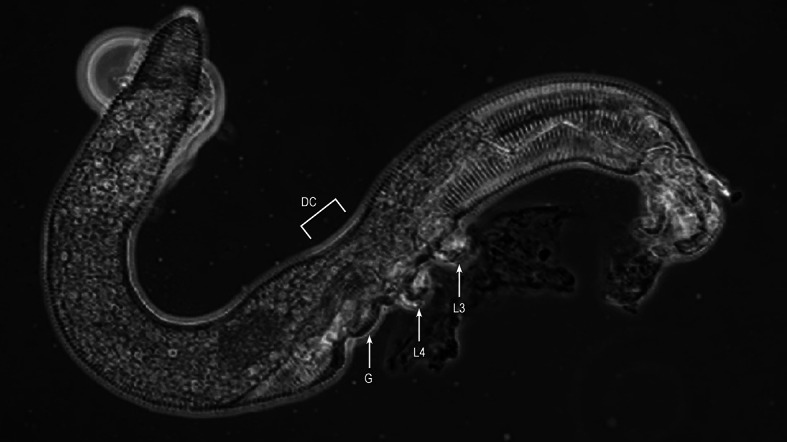
Live specimen of *Osperalycus tenerphagus* showing the constriction of the metapodosomal and genital region—freeze frame taken from video (see Online Resource 4). DC, dorsoventral constriction; G, genital region; L3, leg 3; L4, leg 4

Longitudinal contractions along the main body are obviously essential to locomotion in the Nematalycidae. However, dorsoventral constriction was also observed in the metapodosomal and genital region of *Osperalycus*. The close-up views of live *Osperalycus* (phase contrast microscopy) revealed waves of dorsoventral constriction along the metapodosomal and genital region (Online Resource 4; Fig. 6). During the progression of a wave, the anterior or posterior region of the body extends out longitudinally from the metapodosomal and genital region. Dorsoventral constrictions were not observed anywhere else along the body.

The body appears to be very pliable. When a small area of the idiosoma is flattened by an external force, it can readily re-inflate. This was observed when parts of the live specimens were accidentally squashed while maneuvering them under a stereomicroscope.

## Discussion

### Metapodosomal and genital body region

The results of this study indicate that the peristaltic locomotion of *Gordialycus* may be unique. *Osperalycus*, *Cunliffea* and cf. *Psammolycus* appear to employ a different mode of locomotion that involves dorsoventral constriction and expansion of the metapodosomal and genital region. The longitudinal striations that are present above the legs and genitalia (Fig. 2a, b) allow the lateral integument to fold, therefore enabling the dorsoventral constriction of this body region (Fig. 6). This is the same integumental mechanism by which the annular regions longitudinally contract—the striations move together and the palettes interdigitate. Whereas annular striations allow extension and contraction along a longitudinal axis, longitudinal striations that are located on the side of the body allow expansion and constriction of the dorsoventral axis. The constriction of the metapodosomal and genital region would help to generate the large amount of hydraulic pressure needed to extend the body in annular regions (or the posterior or anterior region), where there is no change in body diameter. This is consistent with the videographic data of Online Resource 4, which shows the extension of the anterior region coinciding with the dorsoventral constriction of the metapodosomal and genital region.

The dorsoventral constrictions of the metapodosomal and genital region are almost certainly effected by dorsoventral muscles. In the annular regions, the longitudinal muscles underlie the integument, forcing the annuli together when they contract. The longitudinal striations probably work in a similar way. Although no attempt has yet been made to observe the muscles of the metapodosomal and genital region, we hypothesize that dorsoventral muscles should be positioned at the sides of this region, just beneath the integument with the longitudinal striations. If the dorsoventral muscles were only near the midline, the integument—which is highly pliable—would likely bend into the body instead of fold around the edge of the body; longitudinal striations would have no obvious function.

Additional hydraulic pressure can also be generated from the center of the body by contracting legs III and IV into the body (Fig. 2d). This could provide the additional advantage of moving the legs out of the way when the center of the body moves through tight gaps. Note that because legs III and IV project downwards, they impede movement by projecting away from the movement path of the main body, making the mite less streamlined. By contrast, legs I and II extend forwards, keeping within the movement path of the main body. Therefore, legs I and II do not appear to impede the movement of the main body when it is moving through tight spaces. They would therefore not be required to contract into the body.

The ability to dramatically alter the volume of the metapodosomal and genital region allows the mite to use the center of its body to generate the pressure needed to extend or contract an annular region of the body. The action of each annular region can therefore be independent of the other, allowing for a relatively versatile form of locomotion. While the annular regions extend or contract, the metapodosomal and genital region can be anchored in place by legs III and IV. But legs III and IV can also be tucked into the body (see above) or used for crawling.

**Fig. 7:**
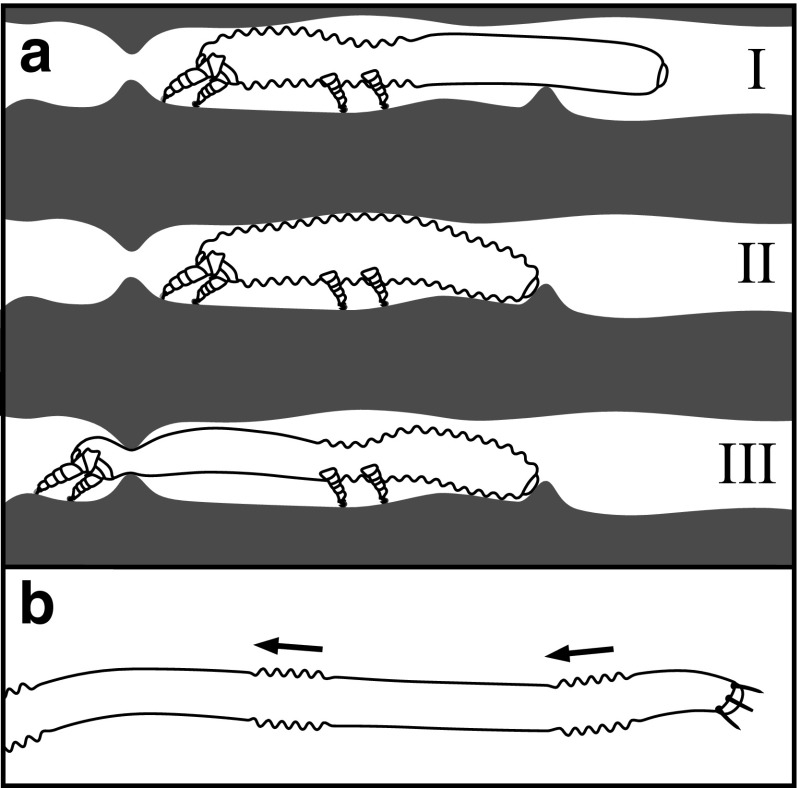
Different modes of locomotion. **a** Hypothesized mode of locomotion for relatively short nematalycids moving through tight spaces.* I–II*: the expansion of the metapodosomal and genital region can allow the posterior region to contract without requiring the anterior region to extend.* II–III*: the constriction of the metapodosomal and genital region can then proceed in conjunction with a well anchored posterior region in order to extend the anterior region of the body through a narrow gap. **b** Peristalsis along the posterior region of *Gordialycus*. Zigzag lines indicate areas that are longitudinally contracted

This mode of locomotion could be especially useful when the mite is attempting to squeeze through tight gaps and crevices. The dorsoventral expansion of the metapodosomal and genital region can enable the posterior region to be maneuvered and anchored into the soil before the anterior region of the mite then extends forwards via the dorsoventral constriction of the metapodosomal and genital region (Fig. 7a). In this way, these mites are able to securely anchor themselves when attempting to squeeze their anterior regions through tight spaces.

*Gordialycus* lacks the longitudinal striations (Fig. 2c) needed to dorsoventrally constrict the metapodosomal and genital region. Instead, this mite is probably only able to move around through peristalsis (Fig. 7b). Longitudinal striations would cause the interruption of peristaltic waves along the length of the body. The dorsoventral expansion of the metapodosomal and genital region would therefore appear to be an impediment to locomotion via peristalsis. With respect to squeezing through tight gaps, the extremely long posterior region would help to anchor the mite in place while the front of the mite extends forwards at the end of a peristaltic wave.

Legs III and IV of *Gordialycus* may be reduced for several reasons. Firstly, their role in anchorage would be less important. Because the metapodosomal and genital region does not constrict or expand dorsoventrally, the legs are not needed to anchor this region of the body in place during the longitudinal contraction or extension of other body regions. Secondly, like the longitudinal striations, the legs would interfere with the completion of peristaltic waves along the length of the body. This is most obvious with respect to the coxal fields, which are all very large in the other genera. In *Gordialycus*, the legs have moved in towards the midline as a result of the dramatic reduction in size of the coxal fields (Fig. 2c). Once legs III and IV become less important with respect to anchorage, there should be strong selective pressure towards reduction. As mentioned above, legs III and IV make the mite less streamlined by projecting away from the movement path. Their reduction would therefore help the locomotion of this region of the body through tight spaces.

In the video footage of *O. tenerphagus*, no peristaltic waves were observed. It could be that peristalsis is reserved for particular situations. Alternatively, it may be that peristalsis is not compatible with the dorsoventral expansion and constriction of the metapodosomal and genital region. Furthermore, it may be that *Gordialycus*, which reaches over 1.0 mm in length, is the only known genus of the Nematalycidae that is long enough to be able to make effective use of peristalsis.

### Anal valves

The anal valves appear to play an important role in locomotion by providing an anchor from which the rest of the body can push off. They may be especially important to the locomotion of the genera with long posterior regions—*Gordialycus* and *Osperalycus* (Fig. 3a, b). The genera with short posterior regions would appear to be able to drag them along.

The videographic data shows that *Osperalycus* is able to move around by pushing off against the tip of its opisthosoma, which is first anchored or wedged into the soil (Online Resource 1). The anal valves (Fig. 3a, b), which protrude out strongly in *Osperalycus* and *Gordialycus*, are appropriately structured and positioned to help anchor the tip of the opisthosoma. It is not known whether the anchorage of the tip is accomplished through a wedging or clasping action.

The anal valves are especially prominent in *Gordialycus* relative to the size of the opisthosomal tip (Fig. 3a). It is likely that they are important in the initiation of peristalsis, which begins at the opisthosomal tip. When a wave of contraction instigates in the longitudinal muscles at the tip of the opisthosoma, the tip is pulled inwards and dislodged from the soil as the associated integument contracts. A subsequent wave of extension would then closely follow, causing the tip to extend outwards until it is lodged into a new position in the soil substrate. The wave of extension is the result of the relaxation of the associated longitudinal muscles in conjunction with hydraulic pressure generated by muscle contraction in other regions. As the wave of extension extends further forwards through the body, the corresponding region of the body initially moves forwards through the soil because it is extending away from the anchored anus. This wave continues to proceed forwards into the contracted portion of the body in front of it, causing peristaltic motion of the entire body (Fig. 7b). The relatively long setae near to the anal valves may be used to locate a suitable anchorage point. The setae along the rest of the opisthosoma are short enough to be barely, if at all, noticeable (Fig. 1a). This is possibly an adaptation for reducing friction during the locomotion of such a long body.

The posterior region of *Osperalycus* is typically dragged around by the protonymph (Online Resource 3). The adult stages of the short bodied genera of Nematalycidae—*Cunliffea* and cf. *Psammolycus*—have a posterior region that is similar in length to that of the protonymph of *Osperalycus*. It is therefore possible that the posterior region is commonly dragged around by all of the active instars of *Cunliffea* and cf. *Psammolycus*. Moreover, the weakly projecting anal valves of *Cunliffea* and cf. *Psammolycus* (Fig. 3c, d) indicate that the anal region has a less significant role with respect to anchorage. However, it is also possible that the anal region is still used to facilitate anchorage under certain circumstances, such as when these mites are squeezing through tight gaps (Fig. 7a).

### Palette shape

Variation in the shape of the palettes between genera may be an adaptive trait that accommodates the significant intergeneric differences in the length of the posterior regions. The ability of these mites to maneuver through tight spaces requires anchoring one region of the body firmly in place while another extends or contracts. The secure placement of the static region is essential to success (Fig. 7a). The short posterior regions of some nematalycids cause them to encounter little overall friction when moving around. For this reason, they are more likely to have problems pertaining to slippage. Pointed palettes may therefore be an adaptation for improving the grip of any region of the body that is placed firmly against a surface. This is consistent with the correlation between the shape of the palettes and the relative length of the body. Nematalycids with short posterior regions—*Cunliffea* and cf. *Psammolycus*—appear to have pointed palettes that increase anchorage (Fig. 5c, d). The shortest genus (cf. *Psammolycus*) has the palettes with the sharpest points.

The more elongated bodies of *Gordialycus* and *Osperalycus* would encounter a lot of friction when contracting, extending or being dragged along. Their mobility depends on waves of extension and contraction along the long posterior regions of their bodies; they would probably not be able to move around using only their legs. Pointed palettes would likely present a serious encumbrance to the locomotion of these mites. Instead they have round palettes that are orientated in the direction of movement, enabling them to slide their long posterior regions over surfaces with relative ease (Fig. 5a, b). Although their integument has less grip or traction, anchorage is facilitated by their longer bodies.

## Conclusion

The Nematalycidae are well adapted for life in the mineral regolith, having originated a number of important adaptations for this habitat. The integument of the Nematalycidae is well suited to movement in tight spaces. These mites can contract one annular region in order to generate the hydraulic pressure needed to extend another. Most significantly, the mode of locomotion and associated morphology appears to be largely determined by the relative degree of elongation. Peristalsis is clearly essential to *Gordialycus*, which is the only known genus of Nematalycidae with an incredibly long body. But peristalsis was not observed in *Osperalycus*, and it may be that the use of peristalsis is restricted to *Gordialycus*. *Cunliffea* and cf. *Psammolycus* are probably too short to be able to make effective use of peristalsis. Instead, these mites appear to use a mode of locomotion that involves the dorsoventral expansion and constriction of their central region.

Interestingly, the functional morphology of *Osperalycus* appears to be transitional between *Gordialycus* and the other genera of Nematalycidae (Table 1). *Osperalycus* resembles *Gordialycus* in having round palettes and a strongly projecting anal vale. The posterior region of *Osperalycus* is much shorter than the posterior region of *Gordialycus* but much longer than the posterior region of the other genera. This mite resembles *Cunliffea* and cf. *Psammolycus* in having a metapodosomal and genital region that can dorsoventrally constrict and expand; this feature is clearly absent in *Gordialycus*.

Peristalsis almost certainly represents a derived mode of locomotion within the Nematalycidae. The primitive mode of locomotion—the dorsoventral expansion and constriction of the central region—provides short bodied nematalycids with a versatile way of contracting and extending annular regions. The transition to peristalsis allowed the evolution of a much longer body length. This is because peristaltic waves allow many short portions of a very long body to be moved along in quick succession; there is no need to extend or contract the entirety of a long body region.

Once the switch to peristalsis was made, the old mode of locomotion was probably not retained for very long as a supplementary form of movement. Instead, key features of the old mode of locomotion were likely to have been quickly jettisoned or modified because they would have interrupted peristaltic motion (viz. large coxal fields and longitudinal striations). Therefore, the successful transition to peristalsis probably manifested as a large scale and dramatic evolutionary event, one mode of locomotion being rapidly and completely replaced by another.

### Electronic supplementary material

Below is the link to the electronic supplementary material.

**Online Resource 1** Anchorage of the posterior region – *Osperalycus tenerphagus* (adult/tritonymph) (adult/tritonymph) (MPG 66444 kb)

**Online Resource 2** Anchorage of the posterior region – *Osperalycus tenerphagus* (protonymph) (MPG 55244 kb)

**Online Resource 3** Dragging of the posterior region – *Osperalycus tenerphagus* (protonymph) (MPG 10524 kb)

**Online Resource 4** Close-up showing the constriction of the metapodosomal and genital region (first clip: normal speed; second clip: quarter speed) – *Osperalycus tenerphagus* (late instar) (MPG 25330 kb)

## Appendix: Material examined under LT-SEM

*Osperalycus* *tenerphagus*—USA, Ohio, Franklin Co., Kinnear Road, 39.9990N–83.0468W, silty clay loam from suburban prairie (including shrubs, grasses and small trees); collector: Samuel Bolton, August-2011, 50 cm deep: Protonymph × 1; Deutonymph × 1; Tritonymph × 6; Adult × 9.

*Gordialycus* sp. A—USA, California, Imperial County, Algodones Dunes, Imperial Sand Dunes Recreation Area, 32.9811N–115.1317W, bottom of sand dune; collector: Samuel Bolton, October-2013, 10 cm deep: larva × 3; nymph/adult × 7.

*Gordialycus* sp. B—USA, Indiana, Lake County, Marquette Park, 41.6156N–87.2743W, top of sand dune; collector: Samuel Bolton, May-2013, 10 cm deep: nymph/adult × 5.

*Cunliffea strenzkei*—USA, Indiana, Lake County, Marquette Park, 41.6156N–87.2743W, top of sand dune; collector: Samuel Bolton, May-2013, 10 cm deep: nymph/adult × 6. USA, Florida, Highlands Co., Highlands Hammock Park, 27.4713N 81.5646W, sandy soil from sparsely wooded area; collector: Samuel Bolton, April-2011, 30 cm deep: tritonymph/adult × 2.

cf. *Psammolycus* sp. n.—USA, Florida, Highlands Co., Highlands Hammock Park, 27.4713N 81.5646W, sandy soil from sparsely wooded area; collector: Samuel Bolton, April-2011, 30 cm deep: post-larva × 2.

Note that all adults were female.
